# Genome-wide identification and analysis of terpene synthase (*TPS*) genes in celery reveals their regulatory roles in terpenoid biosynthesis

**DOI:** 10.3389/fpls.2022.1010780

**Published:** 2022-09-29

**Authors:** Mengyao Li, Xiaoyan Li, Jin Zhou, Yue Sun, Jiageng Du, Zhuo Wang, Ya Luo, Yong Zhang, Qing Chen, Yan Wang, Yuanxiu Lin, Yunting Zhang, Wen He, Xiaorong Wang, Haoru Tang

**Affiliations:** ^1^ College of Horticulture, Sichuan Agricultural University, Chengdu, China; ^2^ Institute of Pomology & Olericulture, Sichuan Agricultural University, Chengdu, China

**Keywords:** celery, terpene synthase (*TPS*) gene, genome-wide analysis, evolution, expression pattern, HS-SPME-GC/MS, terpenoids

## Abstract

Terpenes are an important class of secondary metabolites in celery, which determine its flavor. Terpene synthase (*TPS*) has been established as a key enzyme in the biosynthesis of terpenes. This study systematically analyzed all members of the *TPS* gene family of celery (*Apium graveolens*) based on whole genome data. A total of 39 celery *TPS* genes were identified, among which TPS-a and TPS-b represented the two largest subfamilies. 77 *cis*-element types were screened in the promoter regions of *AgTPS* genes, suggesting the functional diversity of members of this family. Gene Ontology (GO) and Kyoto Encyclopedia of Genes and Genomes (KEGG) enrichment analyses showed that *AgTPS* genes were enriched in multiple terpenoid biosynthesis pathways. Transcript abundance analysis and qRT-PCR showed that most *AgTPS* genes were differentially expressed in different tissues and colors of celery, with *AgTPS 6, 9, and 11* expressed differentially in tissues, while *AgTPS31*, *32*, and *38* are expressed differently in colors. More than 70% of the celery volatile compounds identified by HS-SPME-GC/MS were terpene, and the most critical compounds were β-Myrcene, D-Limonene, β-Ocimene and γ-Terpinene. Principal component analysis (PCA) showed that compounds (E)-β-Ocimene, D-Limonene, β-Myrcene and γ-Terpinene predominantly accounted for the variation. Further correlation analysis between gene expression and terpenoid accumulation showed that the four genes *AgTPS9*, *25*, *31* and *38* genes may have positive regulatory effects on the synthesis of D-Limonene and β-Myrcene in celery. Overall, this study identified key candidate genes that regulate the biosynthesis of volatile compounds and provide the foothold for the development and utilization of terpenoids in celery.

## Introduction

During plant growth, many volatile substances with different biological characteristics and functions are synthesized through primary metabolism and secondary, and these substances are widely involved in a series of activities. Among these, terpenoids are the major constituents of many plant aromas and play an important role in the communication between plants and the environment, plants and animals, and plants and plants (Tholl, 2015; Wang et al., 2020). Although there are many kinds of terpenoids, all compounds are synthesized by a series of pathways by the precursors dimethyl allyl diphosphate (DMAPP) and isoprenyl diphosphate (IPP) (Newman and Chappell, 1999). Terpene synthase (*TPS*) is located at the branch point in the biosynthetic pathway of terpenoids and is the key enzyme for terpenoid biosynthesis (Jia et al., 2022); it can catalyze precursor substances (DMAPP and IPP) to form a variety of terpenoids such as hemiterpenoids (C5), monoterpenes (C10), sesquiterpenes (C15) and diterpenes (C20) (Tholl, 2006). It has been established that fruits are more flavorful due to their terpenoids (Mele et al., 2021), while some terpenoids have bactericidal and insecticidal properties in vanillas and spices and are often used to preserve food (Tassou et al., 2012).

It is well-established that the TPS gene family can be systematically divided into seven subfamilies, namely TPS-a, TPS-b, TPS-c, TPS-d, TPS-e/f, TPS-g and TPS-h. Different subfamily genes encode different functions of terpene synthase, among which TPS-a is primarily responsible for synthesizing sesquiterpene synthase, TPS-b and TPS-g mainly synthesize monoterpenoids, and the three subfamilies belong to the angiosperm-specific classification, TPS-c and TPS-e/f are primarily synthetic diterpenes synthases (Chen et al., 2011). Interestingly, the TPS-c subfamily is thought to be its ancestral branch, containing the *CPS* gene of gymnosperms and angiosperms. TPS-e/f contains gymnosperms and angiosperms *KS* genes, while TPS-d belongs to a subfamily endemic to gymnosperms and also a variety of functions (Bohlmann et al., 1998). Over the years, genome sequencing of multiple species, has led to the excavation of more TPS gene families. For example, *TPS* genes have been identified in *Arabidopsis thaliana* (33 *TPSs*) (Aubourg et al., 2002), *Brassica rapa* (28 *TPSs*) (Yang et al., 2006), *Vitis vinifera* (69 *TPSs*) (Martin et al., 2010), *Solanum lycopersicum* (29 *TPSs*) (Falara et al., 2011), *Eucalyptus globulus* (106 *TPSs*) (Alicandri et al., 2020), and *Mentha longifolia* (73 *TPSs*) (Chen et al., 2021). During evolution, some species have undergone specific expansions in the lineage of their subfamily to adapt to the environment, leading to the divergence of gene function (Jiang et al., 2019). The roles and regulatory mechanisms of terpene synthases in terpenoid synthesis are heterogeneous from for different species. Studies have demonstrated the biological role of terpenoids in plants against insects and adversity; the sesquiterpene (E)-nerolidol and (E)-β-farnesene synthesized by the rice terpene synthase *OsTPS18* can play an important role in defense systems (Kiryu et al., 2018). At the same time, terpenoids are major contributors to flavor in various kinds of fruits; for instance, the monoterpenoid E-geraniol, is an important volatile substance that can significantly contribute to the flavor of sweet orange (Li et al., 2017). Therefore, studying of the role and regulatory mechanism of the terpene synthase (*TPS*) gene in different species can provide a theoretical basis for the resistance mechanism of plant terpenoids under stress conditions and imparting fruit flavor.

Celery (*Apium graveolens* L.) is a biennial vegetable of the Apiaceae family, rich in vitamins, apigenin, cellulose, coumarin, polyphenols and other nutrients and has a unique flavor (Cheng et al., 2021; Li et al., 2022). In addition, celery seeds are often used as food flavoring agents, and their extracts have various medicinal properties such as antioxidant, hypolipidemic, and hypoglycemic (Sowbhagya, 2014; Keilwagen et al., 2017). Due to its high nutritional value and delicious taste, celery is deeply loved by consumers and widely cultivated worldwide. Sellami et al. (2012) only identified 25 volatile compounds in roots and leaves of celery in the form of essential oils. Another study reported 85 volatile compounds in celery seeds, including 20 terpenoids and six phthalates, while limonene is the main component of volatile compounds in celery seeds (Yan et al., 2021). The fresh celery leaves (leaf blades and petioles) are the main edible parts of celery. However, no in-depth study has hitherto been conducted on volatile compounds in fresh celery leaves, and the genes that regulate volatile compounds in celery have not been comprehensively explored.

In this study, all members of the TPS gene family were identified and systematically analyzed from the whole genome of celery, and the specificity of volatile substances in different varieties and tissues of celery was determined by using HS-SPME-GC-MS. Moreover, the TPS gene family members involved in the synthesis of celery terpenes were identified. Our results provided theoretical guidance for the utilization of volatile components of celery and important clues for further elucidating the regulatory role of key genes in the biosynthesis of volatile substances.

## Materials and methods

### Plant materials

Celery varieties ‘Hongcheng Hongqin’, ‘Baigan Yihao’ and ‘Siji Lvxiangqin’, which characterized by red, white and green petioles respectively, were used as plant materials to explore the differences among varieties. For tissue-specific (roots, leaf blades and petioles) studies, ‘Siji Lvxiangqin’ was selected because it is one of most widely green variety in China. The seeds were put into the light incubator for germination, the germinated seeds were planted in a seedling tray and cultivated in an artificial climate chamber. Plants samples (roots, leaf blades and petioles) were harvested until plants were grown to 10 leaves (about 20 cm height). The samples were immediately stored in -80°C after being snap-frozen in liquid nitrogen for RNA extraction and determination of VOCs.

### Screening and identification of TPS genes in celery

Celery protein sequences were downloaded from two published celery genome databases (http://apiaceae.njau.edu.cn/celerydb and http://celerydb.bio2db.com/) (Li et al., 2020; Pei et al., 2021) to build a local blast database. The hidden Markov model (HMM) of the TPS domains (PF01397 and PF03936) were obtained *via* screening celery genome sequences by HMMER software, and the sequences were further submitted to NCBI-CDD and Pfam to confirm the TPS domain. TargetP (http://www.cbs.dtu.dk/services/TargetP/) and WOLF PSORT (https://www.genscript.com/wolf-psort.html) were used for subcellular localization analysis, and ExPASy online tool (http://web.expasy.org/protparam/) was performed to analyze physicochemical properties.

### Sequence alignments and phylogenetic analysis of AgTPS genes

The TPS protein sequences of *Arabidopsis thaliana* and tomato were obtained from TAIR (https://www.arabidopsis.org/) and PlantTFDB (http://planttfdb.gao-lab.org/), respectively. Multiple sequence alignments were made using Clustal W, and MEGA X software was then used to generate a neighbor-joining (NJ) tree for phylogenetic analysis.

### Characterization of conserved motif distributions and structures, and function annotation of AgTPS genes

Using the online MEME tool (http://meme-suite.org/) to predict the conserved motifs of AgTPS proteins, and the size parameters of the conserved motifs was set from 10 to 100 aa with the maximum motif number of 15. The gene structure and conserved motifs of the *AgTPS* genes were plotted using TBtools. The sequences of 2000 bp upstream promoter regions of *AgTPS* genes were selected and submitted analyzed to determine the cis-acting element using online website Plant CARE (http://bioinformatics.psb.ugent.be/webtools/plantcare/html/) for cis-acting element prediction. GO and KEGG functional annotation of AgTPS proteins were performed by PANNZER2 (http://ekhidna2.biocenter.helsinki.fi/sanspanz/) and DAVID (https://david.ncifcrf.gov/), respectively.

### Collinear analysis of TPS gene family in celery

Genome data of carrot were obtained from carrot database (https://phytozome-next.jgi.doegov/pz/portal.html) and coriander database (http://cgdb.bio2db.com/), respectively. Collinear relationships between three Apiaceae species were analyzed by MCScanX software and visualized by TBtools software. Synonymous (Ks) and non-synonymous (Ka) substitutions per site between duplicated TCP genes pairs were subsequently calculated using KaKs Calculator v.1.2 software (Zhang et al., 2006).

### Transcript abundance analysis and quantitative real-time PCR validation expression-specific analysis

Transcriptome sequencing data of celery in differently colored petioles and differently tissues were downloaded from the National Genomic Science Data Center (https://ngdc.cncb.ac.cn/) with the accession number of CRA001997. Based on the transcriptome data, the transcript abundance of *AgTPS* in different samples were calculated using FPKM values and mapped using the Heatmap program in the TBtools.

Total RNA of the celery samples was extracted with an RNA extraction kit (Ai Weidi Biotechnology Co., Ltd., Shenzhen, China). cDNA was synthesized by Goldenstar RT6 cDNA Synthesis Kit (Beijing TsingKe Biotech Co., Ltd., Beijing, China), and dilute cDNA 10-fold with RNase free water before use in qRT-PCR assays. qRT-PCR was performed with the Bio-Rad Real-time PCR System and Bio-Rad CFX Manager. The primers for qRT-PCR were designed using Primer Premier software, and the primer sequences are listed in **Table S1**. Relative gene expression was normalized with *AgACTIN* as an internal control and calculated with 2^−ΔΔCt^ method (Pfaffl, 2001).

### Extraction and analysis of celery volatile compounds using HS-SPME combined with GC–MS analysis

The mixture of leaves and petioles from different varieties was used for the determination of compounds in different colors. The roots, leaves and petioles from celery variety ‘Siji Lvxiangqin’ were used to analyze different tissues. Each celery sample (1.0 g) was placed in a 20 mL headspace bottle, which contained 5 mL of saturated NaCl solution and 1 μL internal standard solution 2-Octanol. The headspace bottle was kept in a heated oscillator (500 rpm) at 60 °C for 15 min, then the DVB/CAR/PMDS solid phase microextraction fiber (SPME fiber, 50/30 μm, Supelco Inc., Bellefonte, PA, USA) was used in headspace to extract for 30 min. After extraction, the SPME fiber was inserted into the inlet of the GC system for thermal desorption in a non-splitting mode (250°C for 5 min).

The injected sample was analyzed using 7890 gas chromatography system equipped with a 5977B mass spectrometer (Agilent Technologies Inc., Santa Clara, CA, USA). The oven temperature program was started at 40°C for 5 min and then warmed to 250°C at a rate of 4°C min^−1^ and kept at 250°C for 5 min. Helium was used as carrier gas at a flow rate of 1 mL min^−1^. Identification of compounds was achieved by comparing the mass spectra with the National institute of standards and technology library (NIST 14), at a match factor of ≥85, and their retention indices (RIs) were compared with literature values. The PCA-X model diagram is drawn from the collated data used SICMA software.

## Results

### Genome−wide identification and characteristics of *TPS* genes in celery

Based on the whole-genome of celery, 39 *TPS* genes were identified and renamed *AgTPS1*~*AgTPS39* (**Table S2**). Analysis of the physicochemical properties of 39 AgTPSs showed that the number of amino acids is ranged from 279 to 852, and the molecular weights from 32595.51 to 97708.36 Da. The theoretical isoelectric point for AgTPSs was between 4.91 (*AgTPS5*) and 7.27 (*AgTPS31*). The hydrophobicity prediction results showed that all GRAVY values were negative, indicating that the AgTPSs existed as hydrophilic proteins. The subcellular localization prediction showed that except for a few genes that were only localized in the cytoplasm or mitochondria, most *TPS* genes were localized in both the cytoplasm and mitochondria.

**Figure 1:**
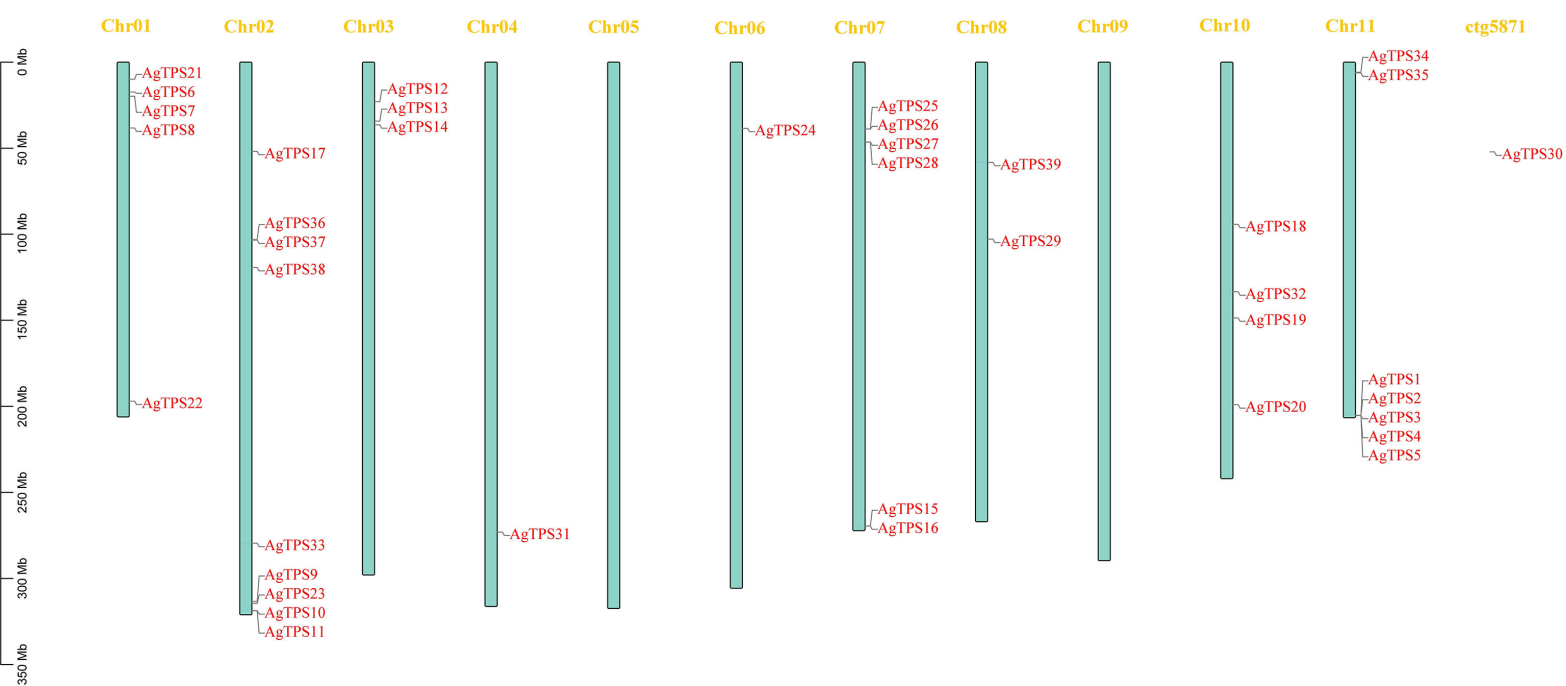
Localization and distribution of celery TPS gene on chromosomes.

Analysis of chromosome distribution showed that except for *AgTPS30*, located on the scaffold, the remaining 38 *AgTPS* genes were distributed irregularly across nine chromosomes, with a maximum of nine genes on Chr02 (**Figure 1**). The *AgTPS* gene was not observed on Chr05 and Chr09. Several *AgTPS* genes were clustered, especially *AgTPS1*, *2*, *3*, *4* and *5* on Chr11. Given that multiple *TPS* genes are reportedly also clustered in tandem on chromosomes in tomatoes (Falara et al., 2011), these genes are widely thought to be associated with tandem duplication events of the TPS gene family.

### Phylogenetic relationship and structure of AgTPS proteins

**Figure 2:**
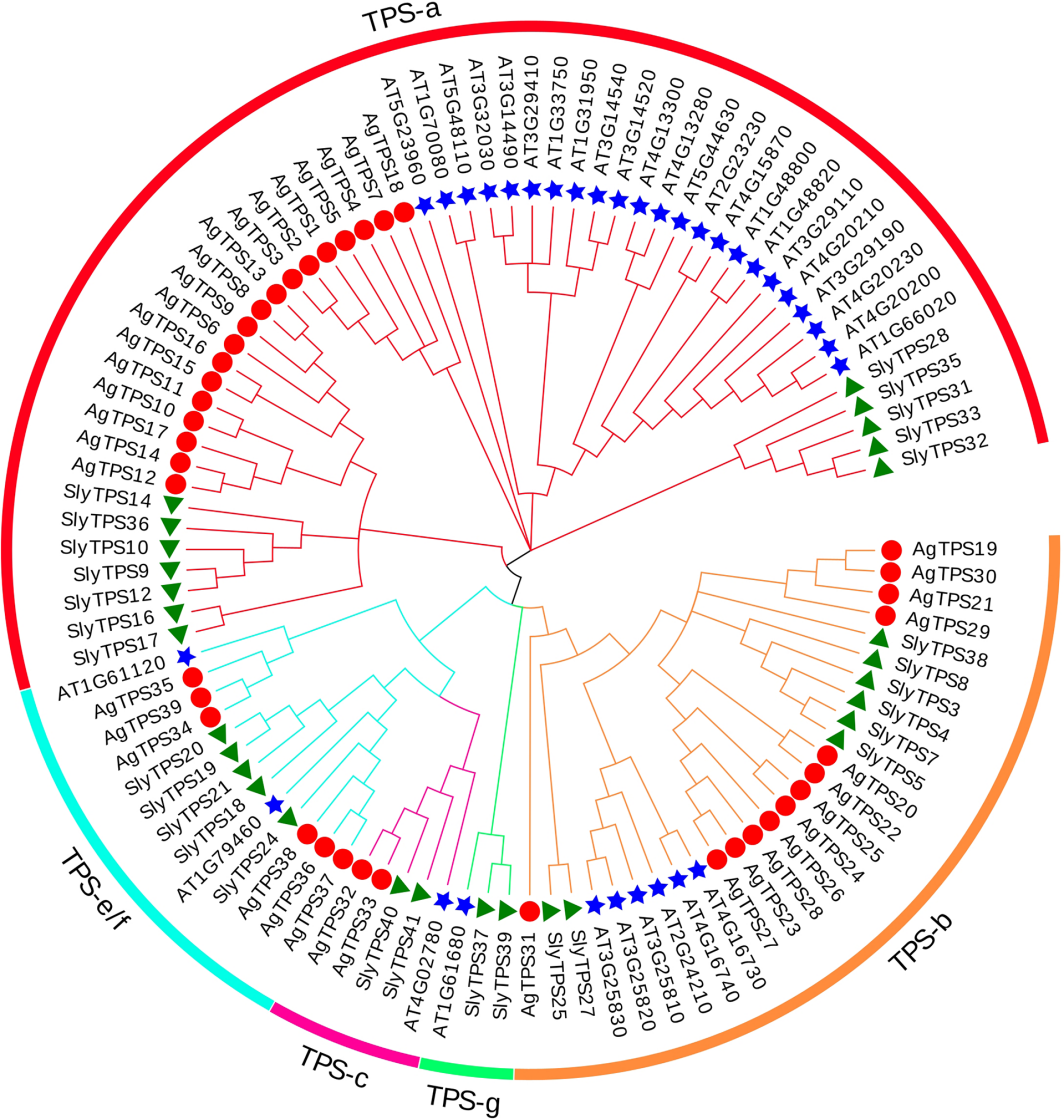
Phylogenetic relationship between celery and other plant TPS proteins. Ag, *Apium graveolens*; Sly, *Solanum lycopersicum*; AT, *Arabidopsis thaliana*.

To classify the AgTPS proteins into subfamilies and identify the evolutionary relationships among celery, arabidopsis and tomato, a phylogenetic tree was constructed using the sequences of TPS proteins from celery (n=39), Arabidopsis (n=33) and tomato (n=29) (**Figure 2**). The results showed that the AgTPSs were divided into four subfamilies of TPS-a, TPS-b, TPS-c, and TPS-e/f, and TPS-a and TPS-b accounted for a relatively large proportion, including 79.5% of *AgTPS* members. TPS-e/f contained only six members, while the TPS-c subfamily contained the least members (n=2).

**Figure 3:**
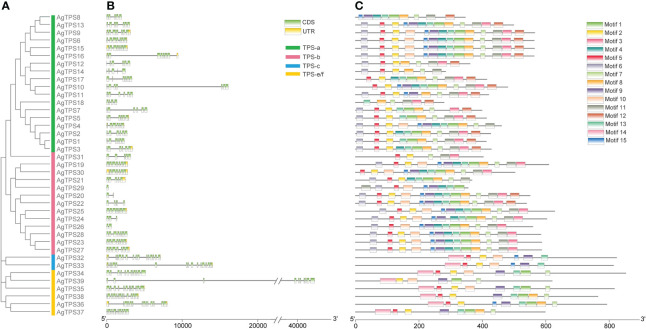
Motif and conserved domain analysis of *AgTPS* genes. **(A)** Phylogenetic evolution of AgTPSs; **(B)** Exon-Intron analysis of *AgTPS* genes; **(C)** Motif analysis of AgTPS proteins in celery.

The exon-intron analysis found fewer exons of TPS-a and TPS-b than those in the TPS-c and TPS-e/f subfamilies; *AgTPS20*, *26* and *29* of the TPS-b subfamily contained only one or two exons, while the *AgTPS33* of the TPS-c subfamily contained up to 15 exons (**Figures 3A, B**). The conserved motifs of AgTPS proteins were analyzed, which showed that Motifs 1-15 were widely distributed in the AgTPS proteins, ranging from 5 (AgTPS31) to 14 (AgTPS6, 9, 15, 16, 20 and 22) (**Figure 3C**). The same motif types and arrangements were observed among members of the same subfamily, with more motifs in TPS-a and TPS-b, and relatively few in TPS-c and TPS-e/f. Motif types and arrangements among members of the same subfamily were the same, with more motifs in TPS-a and TPS-b, and relatively few in TPS-c and TPS-e/f. Motif deletions were present in each subfamily, such as AgTPS12/14 of TPS-a, AgTPS31 of TPS-b, AgTPS32/33 of TPS-c, and AgTPS39 of TPS-e/f, attributed due to the effect of gene transcription post-modification. Motifs 2, 5, 6, 7 and 13 co-existed in more than 23 AgTPSs, indicating that these motif types were highly conserved in the TPS structure.

### Evolution and distribution of TPS family in different varieties

**Figure 4:**
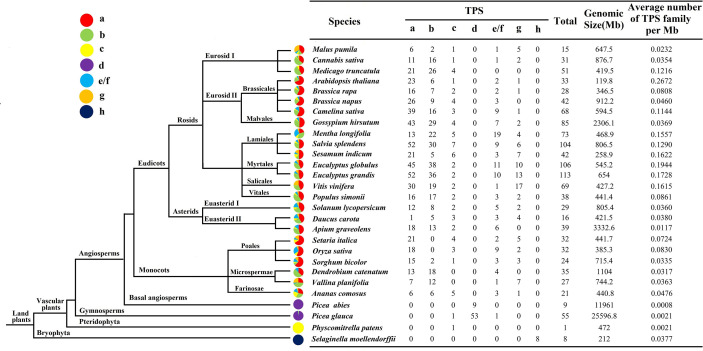
Schematic diagram of the phylogenetic relationships of TPS in different species The evolution and distribution of the TPS family in different species were compared. Each color represents a TPS subfamily, and the colored part represents the proportion of that subfamily.

To gain a deeper understanding of the evolution of the TPS family, the number of TPS in 28 higher plants was analyzed (**Figure 4**). In all species, the density of *TPS* genes was the highest in *Arabidopsis* (0.2672/Mb), followed by *Eucalyptus globulus* (0.1944/Mb) and *V. vinifera* (0.1615/Mb). Except for TPS-d and TPS-h, which only exist in gymnosperms (*Picea abies* and *Picea glauca*) and *Selaginella moellendorffii*, respectively, the TPS of other plants were almost all distributed in TPS-a, TPS-b, TPS-c, TPS-e/f and TPS-g. Interestingly, monocotyledonous plants such as Orchids and Bromeliads did not contain TPS-c members, while Gramineae was mostly distributed in TPS-a, while TPS-b expression was the least. The numbers of *Mentha longifolia* and *V. vinifera* in the TPS-e/f subfamily are 19 and 17, respectively, which are much higher than other species in the same subfamily, which indicates that expansion of this subfamily occurred during plant evolution. It has been found that in monocotyledons and dicotyledons, most of the identified TPS belonged to the two subfamilies of TPS-a and TPS-b, and few were attributed to TPS-g.

### Colinear and evolutionary analysis of carrots, celery and coriander in apiaceae crops

**Figure 5:**
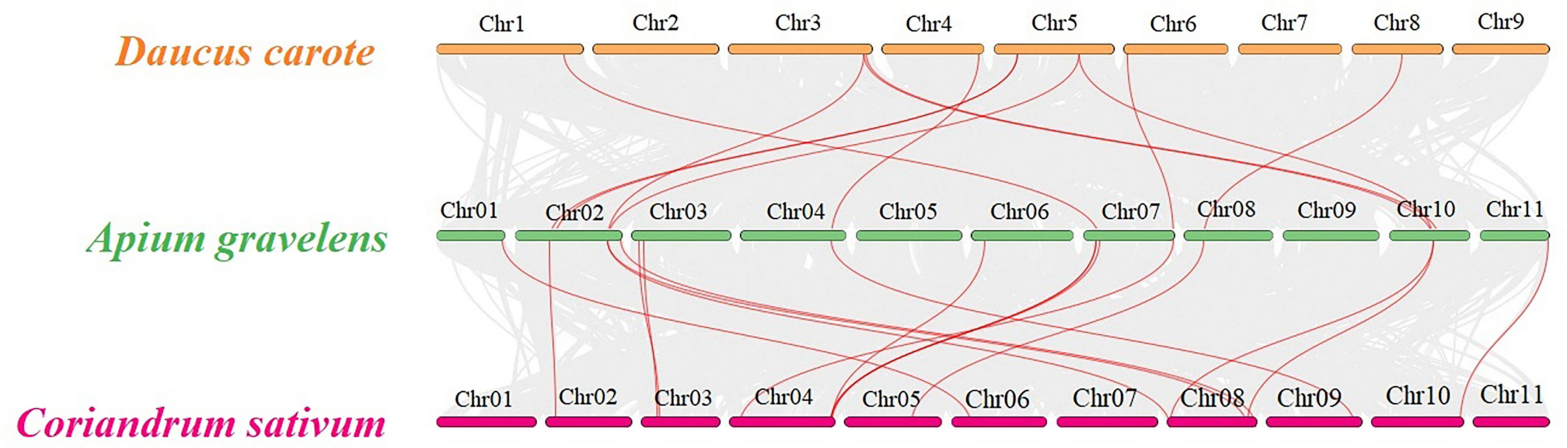
Family collinear analysis of *TPS* genes in carrot, celery and coriander The gray lines represent the collinear relationship of genes between celery/carrot and celery/coriander; the red lines represent the collinear relationship of the TPS gene between celery/carrot and celery/coriander.

Three representative vegetable crops of the Apiaceae family, carrots, celery and coriander, underwent collinearity analysis to explore their evolutionary relationship. 11 TPS paralogous gene pairs were found in celery/carrot and 15 TPS paralogous in celery/coriander (**Figure 5**). The Ka, Ks and Ka/Ks ratios for each paralogous gene pair in celery/celery, carrot/celery and coriander/celery were calculated (**Table S3**), the result showed that Ka and Ks were between 0.01-0.76 and 0.04-1.11, respectively, and all Ka/Ks ratios were less than 1, indicating that the TPS genes in celery, carrot and coriander underwent strong purifying selection in the evolutionary process.

### *Cis*-acting element analysis in the promoter of *AgTPS* genes

**Figure 6:**
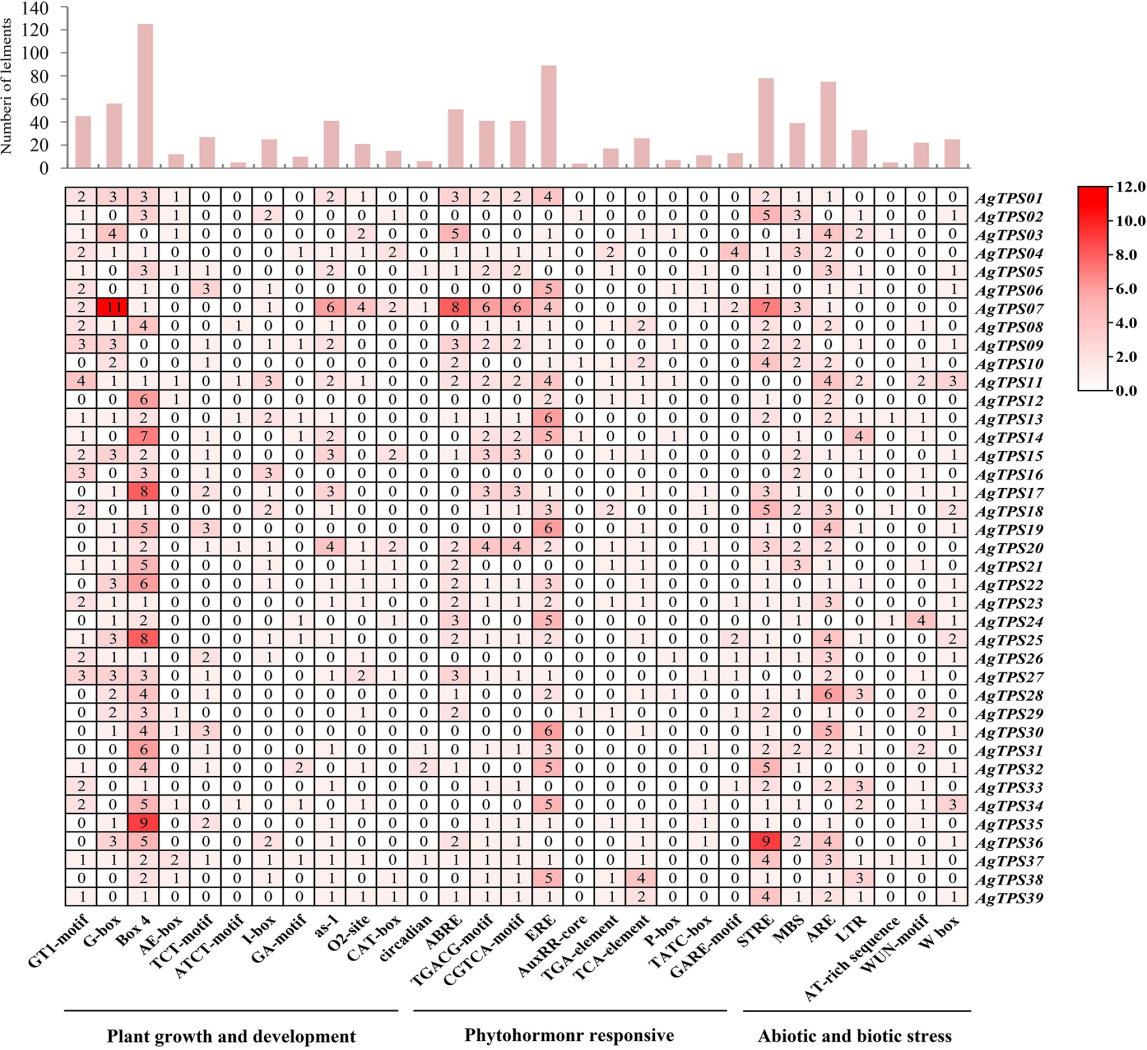
*Cis*-acting elements in the promoter regions of *AgTPS* genes.

To explore the potential biological role of *AgTPS* genes, the cis-element in *AgTPS* promoter regions was analyzed. 77 cis-acting element types were identified and related to plant growth and development (n=48), phytohormone response (n=10) and abiotic and biotic stress (n=19) (**Figure 6** and **Table S4**). The light response (Box4, G-box and I-box), meristem expression elements (CAT-box) and plant circadian regulation elements were highly enriched in the promoters of which Box4, and G-box elements appeared 125 and 56 times, respectively. Some hormone elements were also enriched, such as abscisic acid response element (ABRE) and ethylene response element (ERE), suggesting the *TPS* gene may be involved in various signaling pathways of plant hormones. The ERE element appeared up to 89 times, and 30 *AgTPS* genes were found to contain this element, indicating that *AgTPS* genes may have an extremely important role in promoting ethylene synthesis. Among the cis-element response to abiotic and biotic stress, stress response elements (STRE, n=78) were found in multiple *AgTPS* genes, as well as wound responsive element (WUN-motif, n=22) and wounding and pathogen response element (W box, n=25). Various elements were observed in the promoter regions of the *AgTPS* genes, suggesting that *AgTPSs* may be involved in various biological processes.

### Functional annotation of *AgTPS* gene

**Figure 7:**
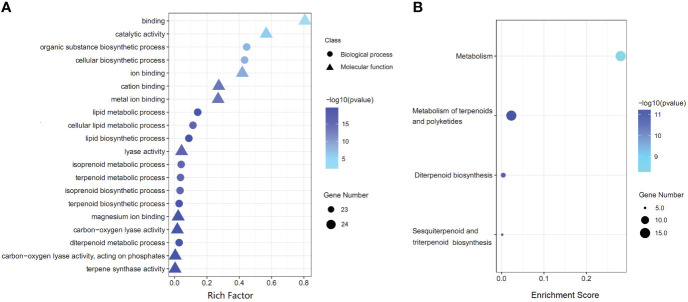
Functional annotation of *AgTPS* genes. **(A)** The top 20 of GO enrichment function annotation. **(B)** KEGG pathway enrichment analysis.

GO functional analysis showed that 34 *AgTPS* genes were enriched into 147 GO terms (p-value<0.05) in three main categories: biological process (BP, 110 terms), cell component (CC, 6 terms), and molecular function (MF, 31 terms) (**Table S5**). The first 20 GO terms with the most significant enrichment are illustrated in **Figure 7A**, where “terpene synthase activity” was the most significantly enriched (24 *AgTPS* members), while “terpenoid metabolic processes” and “terpenoid biosynthesis processes” were also significantly enriched. KEGG enrichment analysis showed that 34 *AgTPS* genes were significantly enriched into four KEGG pathways (p-value<0.05) (**Figure 7B**). Among them, 15 *AgTPS* genes were enriched in the “metabolism of terpenoids and polyketones” pathway, and 11 *AgTPS* genes were enriched in the “sesquiterpene biosynthesis”, “diterpenoid biosynthesis” and “triterpenoid biosynthesis” pathways. These annotations indicate that the *AgTPS* gene is involved in the synthesis of multiple terpenoids.

### Gene expression analysis of *AgTPS* genes in different tissues and varieties of celery

**Figure 8:**
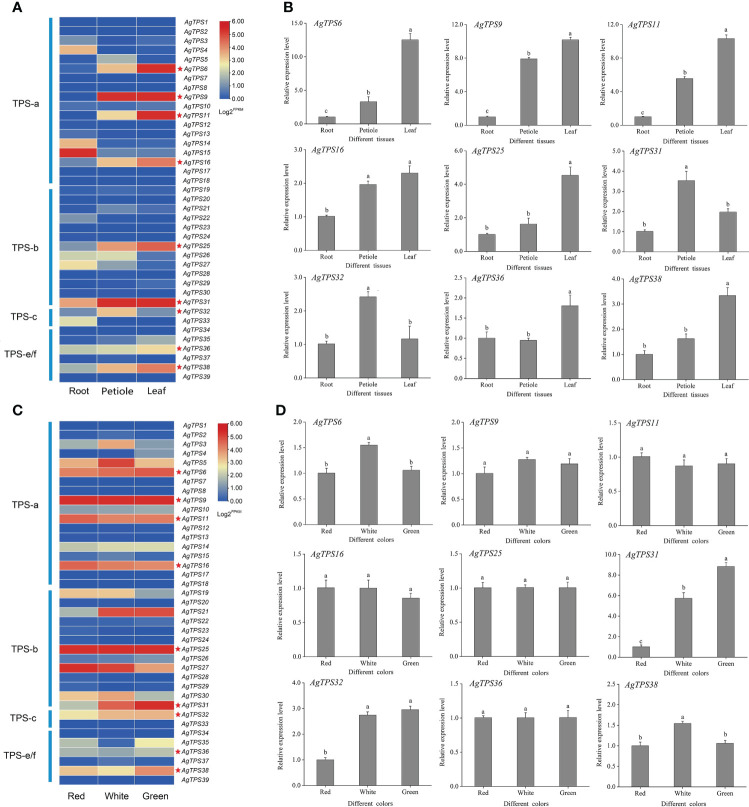
Expression analysis of *AgTPS* family members in different tissues and different varieties. **(A)** Transcriptional expression abundance of *AgTPS* gene in different tissues. **(B)** qRT-PCR results of nine higher-level expressed *AgTPS* genes in different tissues. **(C)** Transcriptional expression abundance of *AgTPS* gene in different colors. **(D)** qRT-PCR results of nine higher-level expressed *AgTPS* genes in different colors.

The transcript abundance of *AgTPS* genes was analyzed based on published transcriptome data in different tissues (roots, petioles, and leaves) and varieties (celery varieties with red, white, and green petioles) of celery. All genes were expressed in the transcriptome samples but exhibited different transcript abundance levels (**Figures 8A**, **8C**). Most *AgTPSs* exhibited higher expression in leaves and petioles than in roots, suggesting that many *AgTPS* genes exhibit tissue specificity. The expression abundance of *AgTPS* genes in different colors of celery showed that most genes of the TPS-a and TPS-b subfamily were expressed in white, green and red celery, while the expression of TPS-c and TPS-e/f subfamilies was relatively low, indicating that there were functional differences between different subfamily members of the TPS gene family in different celery varieties.

For transcriptome validation, nine genes with high expression levels for *AgTPS6*, *9*, *11*, *16*, *25*, *31*, *32*, *36* and *38* were selected for validation by qRT-PCR. The expression profiles of *AgTPS* genes were significantly different in roots, leaves, and petioles, and most *AgTPS* genes showed higher expression in leaves and petioles than in roots (**Figure 8B**). In particular, the expression levels of *AgTPS6*, *9*, and *11* in leaves were about 5-fold and 10-fold higher than in petioles and roots, respectively, suggesting that these genes have obvious tissue specificity. However, differentially expressed TPS genes in different varieties were *AgTPS6*, *31*, *32*, and *38*, and the difference in gene expression of *AgTPS31* was most significant (**Figure 8D**). Compared with red variety, the expression level of *AgTPS31* were about 9-fold and 5-fold in green and white celery varieties, respectively. Members of TPS family exhibited different expression patterns, which confers the functional uniqueness among the family members. Overall, the heterogeneity in expression across different tissues was more significant than across varieties, and qRT-PCR results were consistent with the results of transcriptome analysis.

### Identification of volatile components of celery using SPME–GC–MS

**Figure 9:**
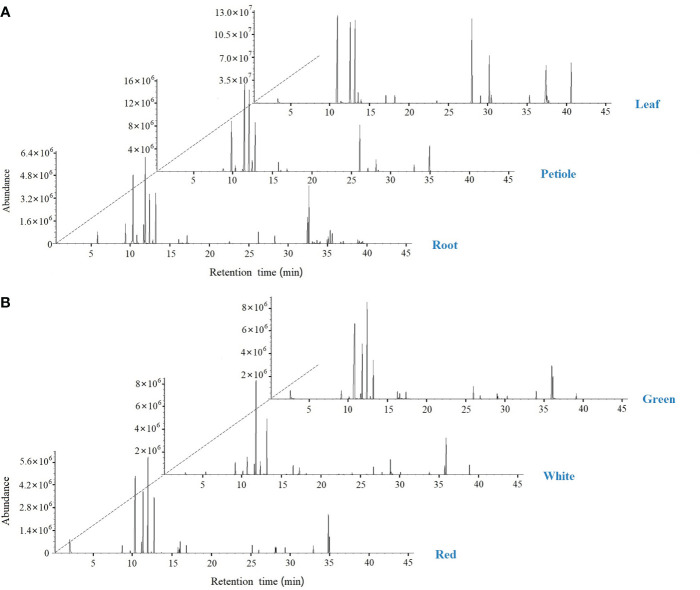
SPME-GC-MS total ion flow chromatogram in celery. **(A)** GC-MS peak plot of roots, petioles, and leaf; **(B)** GC-MS peak plot of red, white and green celery.

The volatile composition of celery in three different varieties and three different tissues (root, petiole and leaf) was determined by HS-SPME-GC-MS based on the NIST14 library and reference compounds. A total of 120 volatile organic compounds and 39 rich compounds were identified (**Figure 9**, **Table 1** and **Table S6**). We found that green celery contained the most volatile compounds, followed by white and red celery. The volatile components of celery were mainly composed of Terpenes, Esters and Phthalide, of which the relatively high contents are β-Myrcene, D-Limonene, (E)-β-Ocimene, γ-Terpinene and Caryophyllene, accounting for more than 70% of the total volatile components. The content of these compounds in the petioles and leaves was much higher than in roots, indicating that terpenoids are mainly concentrated in edible petioles and leaves. Moreover, the leaves and petioles contained the most volatiles and were mostly absent in the roots. However, it was also found that Pentadecane, Bicyclo[4.1.0]heptane, -3,7, 7-T3, and Phenol, 2, 6-bis(1,1-dimethylethyl)-4-(1-methoxyethyl)- appeared only in the roots.

**Table 1:** The volatile compounds and approximate quantities identified in the headspace of celery using SPME-GC-MS harvested at three different colors and tissues.

Compound^a^	Root^b^		Petiole	Leaf	Red^c^	White	Green	CAS^d^
**Alkanes**								
Nonane	6.61 ± 0.42a		4.94 ± 0.28a	0.36 ± 0.02b	1.44 ± 0.16ab	5.96 ± 0.25a	1.93 ± 0.08ab	000111-84-2
Cyclohexasiloxane, dodecamethyl-	0.29 ± 0.02b		3.29 ± 0.27a	2.91 ± 0.16a	1.84 ± 0.08ab	1.62 ± 0.27ab	2.54 ± 0.30a	000540-97-6
Cymene	10.63 ± 1.01ab		4.53 ± 0.48b	0.11 ± 0.01c	11.60 ± 0.42ab	17.94 ± 0.67a	8.54 ± 0.41ab	025155-15-1
Bicyclo[4.1.0]heptane,-3,7,7-T	3.19 ± 0.02a		0 ± 0b	0 ± 0b	0 ± 0b	0 ± 0b	0 ± 0b	018968-23-5
Pentadecane	0.68 ± 0.08a		0 ± 0b	0 ± 0b	0 ± 0b	0 ± 0b	0 ± 0b	000629-62-9
**Total**	21.40 ± 1.55a		12.76 ± 1.03ab	3.38 ± 0.19b	14.88 ± 0.66ab	25.52 ± 1.19a	13.01 ± 0.79ab	
**Aldehydes**								
2-Hexenal	0 ± 0b		3.68 ± 0.34a	2.01 ± 0.22a	0 ± 0b	5.79 ± 0.31a	0 ± 0b	000505-57-7
(E)-2-Hexenal	0 ± 0b		0.03 ± 0b	5.52 ± 0.33ab	19.58 ± 1.00a	0 ± 0b	19.89 ± 0.87a	006728-26-3
Decanal	0 ± 0b		0 ± 0b	0 ± 0b	0 ± 0b	0.53 ± 0.04a	0 ± 0b	000112-31-2
2,4-Heptadienal, (E,E)-	0 ± 0b		0 ± 0b	0 ± 0b	0.52 ± 0.08a	0 ± 0b	0 ± 0b	004313-03-5
**Total**	0 ± 0c		3.71 ± 0.34b	7.53 ± 0.55b	20.10 ± 1.08a	6.32 ± 35b	0 ± 0c	
**Ketones**								
Cyclohexanone	0 ± 0b		0.27 ± 0.06a	0.34 ± 0.05a	0 ± 0b	0.46 ± 0.07a	0 ± 0b	005948-04-9
**Terpenes**								
α-Pinene	0.51 ± 0.02b		3.21 ± 0.82a	0 ± 0c	1.81 ± 0.36ab	1.29 ± 0.38ab	1.67 ± 0.12ab	002437-95-8
β-Pinene		11.79 ± 0.80a	6.71 ± 0.36ab	0.96 ± 0.05b	0 ± 0c	0 ± 0c	11.93 ± 5.42a	000127-91-3
β-Myrcene		38.89 ± 0.61b	85.51 ± 0.81ab	164.69 ± 2.74a	3.56 ± 0.33c	6.59 ± 0.62c	146.32 ± 0.43a	000123-35-3
D-Limonene		48.54 ± 0.54b	187.69 ± 1.21a	119.30 ± 0.64ab	63.51 ± 3.20b	163.94 ± 2.47a	84.49 ± 2.98b	005989-27-5
(Z)-β-Ocimene		2.12 ± 0.29b	12.47 ± 1.32a	9.61 ± 0.49ab	0 ± 0c	3.02 ± 0.59b	18.29 ± 1.21a	013877-91-3
(E)-β-Ocimene		23.41 ± 0.53b	159.21 ± 2.21a	115.55 ± 2.91ab	100.81 ± 3.59ab	17.66 ± 0.55b	164.35 ± 3.29a	003779-61-1
β-selinene		0.92 ± 0.05c	13.75 ± 2.01ab	15.28 ± 2.21ab	6.07 ± 0.51b	22.40 ± 1.41a	10.72 ± 0.52ab	017066-67-0
γ-Terpinene	24.72 ± 0.82ab		64.32 ± 0.89a	3.69 ± 0.37b	54.55 ± 1.06a	78.33 ± 0.75a	55.83 ± 1.96a	000099-85-4
α-Terpinene	0 ± 0b		0 ± 0b	0 ± 0b	0.39 ± 0.03a	0 ± 0b	0 ± 0b	000099-86-5
Caryophyllene		6.39 ± 0.37b	60.08 ± 0.93ab	107.86 ± 4.41a	3.95 ± 0.13b	12.71 ± 0.96b	5.84 ± 0.39b	017627-40-6
Camphene		4.22 ± 0.24b	13.92 ± 1.01ab	10.29 ± 0.82ab	11.49 ± 0.59ab	22.21 ± 1.59a	9.50 ± 0.02ab	000079-92-5
2-Carene	0.32 ± 0.09a		0 ± 0b	0 ± 0b	0 ± 0b	0 ± 0b	0 ± 0b	000554-61-0
Humulene	1.00 ± 0.15b		4.05 ± 0.12ab	7.52 ± 0.29a	0 ± 0c	4.17 ± 0.18ab	0 ± 0c	006753-98-6
Neophytadiene	0 ± 0c		1.64 ± 0.08b	39.31 ± 0.79a	1.20 ± 0.15b	13.98 ± 1.47ab	7.89 ± 0.34ab	000504-96-1
1,3,8-p-Menthatriene	0 ± 0b		0.15 ± 0.01a	0.16 ± 0.01a	0.84 ± 0.08a	0 ± 0b	0.76 ± 0.07a	018368-95-1
Terpinolene	0 ± 0b		0.87 ± 0.09a	0 ± 0b	0 ± 0b	0 ± 0b	0.33 ± 0.04a	005975-30-4
5-Pentylcyclohexa-1,3-diene	0 ± 0b		12.01 ± 1.21a	11.04 ± 0.91a	9.93 ± 0.38a	10.31 ± 1.32a	10.52 ± 0.88a	056318-84-4
2,4,6-Octatriene, 2,6-dimethyl-, (E,Z)-	2.48 ± 0.57b		10.24 ± 1.19a	7.39 ± 0.82ab	6.71 ± 1.38ab	1.78 ± 0.39b	12.09 ± 1.26a	007216-56-0
2-methyl-5-pentylcyclohexa-1,3-diene	4.76 ± 0.42b		3.35 ± 0.45b	7.51 ± 0.37ab	8.93 ± 0.38ab	16.31 ± 1.00a	11.67 ± 0.88a	113768-31-3
2,6-dimethyl-2,4,6-octatriene	2.48 ± 0.41b		10.52 ± 0.35a	7.50 ± 0.38ab	1.82 ± 0.06b	9.28 ± 1.34a	14.19 ± 1.56a	003016-19-1
**Total**	172.58 ± 5.91b		649.70 ± 15.07a	627.66 ± 18.21a	275.55 ± 12.23ab	383.98 ± 15.02ab	566.39 ± 21.37a	
**Phthalide**								
Benzene, 1,2,4-trimethyl-	0 ± 0b		0 ± 0b	0 ± 0b	0 ± 0b	0.88 ± 0.03a	0 ± 0b	000095-63-6
Benzene,1,4-dichloro-	0 ± 0b		0.84 ± 0.05a	0.74 ± 0.17a	0 ± 0b	0 ± 0b	1.24 ± 0.08a	000106-46-7
Phenol,2,6-bis(1,1-dimethylethyl)-4-(1-methoxyethyl)-	27.78 ± 0.29a		0 ± 0b	0 ± 0b	0 ± 0b	0 ± 0b	0 ± 0b	005456-18-8
Z-Butylidenephthalide	0 ± 0b		0 ± 0b	0.97 ± 0.02a	0 ± 0b	0 ± 0b	0.74 ± 0.05a	072917-31-8
Naphthalene	4.76 ± 0.41b		16.0 ± 0.42ab	61.90 ± 0.81a	4.27 ± 1.12b	3.29 ± 1.28b	55.92 ± 3.26a	000091-20-3
3-Butylisobenzofuran-1(3H)-one	2.91 ± 0.25b		8.92 ± 1.33ab	15.28 ± 2.19a	10.14 ± 1.59a	5.95 ± 0.32b	14.22 ± 1.48a	006066-49-5
**Total**	35.45 ± 0.95ab		25.76 ± 1.80ab	78.89 ± 3.19a	14.41 ± 2.71b	6.83 ± 1.63b	72.12 ± 4.87a	
**Esters**								
Senkyunolide G	5.57 ± 0.41c		0 ± 0d	63.19 ± 0.01a	18.20 ± 0.06b	66.22 ± 2.27a	36.35 ± 1.56ab	124815-25-4
Senkyunolide I	0 ± 0c		48.17 ± 0.70ab	75.55 ± 2.18a	45.96 ± 2.66ab	16.30 ± 1.09b	68.41 ± 3.35a	094596-28-8
**Total**	5.57 ± 0.41c		48.17 ± 0.70b	138.74 ± 2.19a	64.16 ± 2.72ab	82.52 ± 3.36ab	104.76 ± 4.91a	
**Oxide**								
(Z)-Limonene oxide	0 ± 0b		15.65 ± 0.10a	0 ± 0b	12.99 ± 0.99a	14.34 ± 0.82a	8.94 ± 0.27a	004959-35-7

^a^Volatile compounds were collected by mixture of 1 uL 2-Octanol (internal standard) and 5 mL saturated calcium chloride, while the estimated qualities were calculated on the base of 2-Octanol content; internal standard was used to normalize chromatograms;

^b^Different tissues of celery samples are selected from the more mainly planted green varieties, ‘Siji Lvxiangqin’;

^c^Different colors of celery samples is a mixture of leaves and petioles of edible parts; Red: ‘Hongcheng Hongqin’; White: ‘Baigan Yihao’; Green: ‘Siji Lvxiangqin’;

^d^The CAS number represents a unique numeric identification number for the compound.

### PCA of terpenoids and correlation analysis with *AgTPS* genes

**Figure 10:**
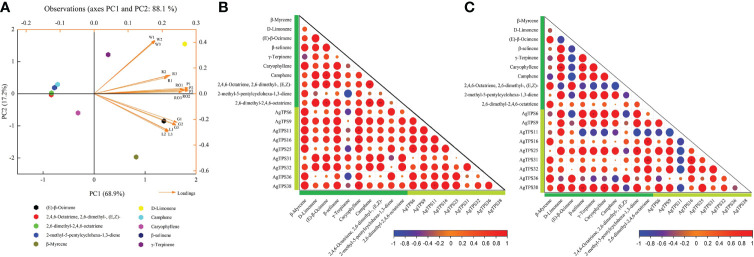
PCA of 10 terpenoids and correlation analysis with *AgTPS* genes. **(A)** Principal component analysis of 10 representative terpenoids (elected from **Table 1**) and six samples; W: white celery; G: green celery; R: red celery; RO: root; P: petiole; L: leaf; **(B)** Correlation between 10 terpenoids and nine *AgTPS* genes in different tissues; **(C)** Correlation between 10 terpenoids and nine *AgTPS* genes in different colors; dark green represent compounds and light green represent genes.

Principal component analysis (PCA) was used to explore the overall distribution and intrinsic variation of volatiles in different tissues and varieties of celery. Ten representative terpenoid volatiles were selected as variables for PCA, and their correlation with nine *AgTPS* genes with higher expression abundance was further analyzed. Based on SIMCA, ModX analysis was performed on six celery samples, and the results showed that the weighted residuals of green celery were the highest among the three celery varieties, petiole and leaf leaves in all three tissues was higher than that of the roots. X/Y Overview Plot PCA for ten compounds indicates that β-Myrcene, D-Limonene, β-selinene and Camphene were well modeled (R2/Q2≥0.5) (**Figure S1**). As shown in **Figure 10A**, 10 volatiles were distributed in different quadrants, and PC2 accounted for 17.2% of the total variance. Significant differentiation was observed among six samples, red and white varieties, as well as roots; petioles were distributed in the upper right quadrant, while green celery leaves were distributed in the lower right quadrant, which indicates that volatiles exhibited significant variations with different colors and parts of celery and may be determined by the environment or genes. Moreover, we found that (E)-β-Ocimene could most likely affect the variation in compound content, followed by D-Limonene, β-Myrcene and γ-Terpinene. There were significant correlations between terpenoids and genes in different varieties and tissues (**Figures 10B, C**). Among them, *AgTPS11*, *16* and *38* were positively correlated with Caryophyllene, and D-Limonene, β-selinene and Caryophyllene were positively correlated with *AgTPS38* and negatively correlated with *AgTPS11*. We also found significant correlations among several terpenoids, such as Caryophyllene and D-Limonene, indicating a correlation in compounds biosynthesized and accumulated among these terpenoids. In addition, *AgTPS11* was negatively correlated with multiple *AgTPS* genes, which suggests that the *AgTPS* gene may play a different regulatory role in terpenoids in different colors of celery. Combined with the above expression results and correlation analysis, we found that the four genes *AgTPS9*, *25*, *31* and *38* have a positive regulatory effect on the synthesis of D-Limonene, β-Myrcene and Caryophyllene.

## Discussion

In this study, a total of 39 *TPS* genes were identified in the celery genome, and the number of *AgTPS* genes was more numerous than genes identified from Arabidopsis (n=32) (Aubourg et al., 2002), tomato (n=29) (Falara et al., 2011) and carrot (n=16) (Song et al., 2020). TPS-a and TPS-b are the two subfamilies with the largest number of *AgTPS* genes in celery, and this distribution is consistent across several species, including *Salvia splendens* (Chen et al., 2021), alfalfa (Allen et al., 2019), and poplar (Irmisch et al., 2014). In addition, six *AgTPS* genes belonged to the TPS-e/f subfamily, but this family has fewer genes in other species, such as carrot (n=3) (Song et al., 2020), cabbage (n=1) (Yang et al., 2006) and apple (n=1) (Nieuwenhuizen et al., 2013), indicating that this subfamily experienced gene replication events during celery evolution. Gene duplication is an important evolutionary mechanism that provides a source of genetic material for species specificity (Magadum et al., 2013). The number of TPS members in celery was relatively high, suggesting that these members play an important role in functional gene diversity, family evolution, and plant adaptation. In the present study, promoter analysis unearthed 77 cis-element types that involves multiple biological processes such as light reactions (Box4, I-box and G-box), phytohormone response (ABRE and ERE), and abiotic or biotic stresses (W box and ARE). Studies found that the biosynthesis of ethylene in bananas may positively or negatively affect the synthesis of terpenes. Moreover, the promoter region of the watermelon TPS gene contains many cis-acting elements associated with phytohormone response, such as ABRE, while overexpression of *ClTPS3* in Arabidopsis could significantly improve the salt tolerance of the transgenic plants (Yuan et al., 2021). Analysis of the cis-elements revealed functional diversity among the various AgTPS family members in celery.

GO and KEGG enrichment analyses showed that the *AgTPS* genes were enriched in multiple terpenoid biosynthetic pathways, suggesting that this family may play key roles in regulating terpenoid synthesis. To explore the regulatory role of *AgTPS* gene in celery, the expression patterns of the *AgTPS* gene in different tissues and varieties of celery were analyzed based on RNA-seq and qRT-PCR. The results showed that most *AgTPS* genes were significantly expressed, especially *AgTPS9*, *25*, *31* and *38*, in different varieties and tissues. It has been shown that the *TPS25 and TPS27* genes can directly regulate terpenes accumulation in tomatoes and can also be obtained indirectly by additional enzyme modification products (Zhou and Pichersky, 2020). *DcTPS04* can regulate the synthesis of D-limonene and β-pinene, and *DcTPS54* regulates the formation of shabilene volatiles in carrots (Reichardt et al., 2020).

HS-SPME-GC-MS was used to detect volatile compounds of different celery varieties and different tissues. The results showed that the volatile compounds in celery mainly consisted of terpenes (70%). Among them, the most critical compounds were β-Myrcene, D-Limonene, β-Ocimene, and γ-Terpinene, consistent with the literature (Tu and Qin, 2017; Yan et al., 2021; Turner et al., 2021a; Turner et al., 2021b). PCA analysis of 10 significantly different terpenoids found that (E)-β-Ocimene, D-Limonene, β-Myrcene and γ-Terpiene were most likely to affect the compound content indicating that the difference in content between these four compounds led to differences in celery terpenoid compounds across different parts and different varieties. Current evidence suggests that terpenoids are also involved in plant defense responses (Zulak and Bohlmann, 2010), and volatile substances D-Limonene and β-Myrcene in celery can reportedly repel whitefly (Tu and Qin, 2017). In this study, D-Limonene and β-Myrcene accumulated significantly in celery, especially green celery with the highest content in leaves and petioles.

To deeply understand the relationship between celery *TPS* gene and terpenoids, correlation analysis was carried out on the compound and *AgTPS* gene, and a definite association was found between gene expression and the terpenoid compound accumulation. Studies on horticultural plants have reported that the *TPS* gene can regulate the type and content of terpenoids in plants. Linalool is a key compound in ripe peaches, and the expression of *PpTPS1* and *PpTPS3* can increase the content of linalool. (Wei et al., 2022). *MrTPS3* and *MrTPS20* in arbutus are key genes that regulate β-caryophyllene and α-pinene synthesis, respectively (Wang et al., 2022). In our study, we predicted four genes *AgTPS9*, *25*, *31* and *38*, may be involved in regulating D-Limonene and β-Myrcene biosynthesis in celery. The results of the present study not only provide a basis for further development of the potential of celery volatiles in the future but also lays the foundation for further study of the function of the TPS gene family in the future.

## Data availability statement

The datasets presented in this study can be found in online repositories. The names of the repository/repositories and accession number(s) can be found in the article/**Supplementary Material**.

## Author contributions

ML and HT contributed to conception and design of the study. XL, JZ, YS, JD, ZW, YTZ, YXL, and YW performed the experiments. XL, YZ, YL, QC, and WH organized the database. ML, XL, and JZ wrote the paper. XW and HT revised the paper. All authors contributed to manuscript revision, read, and approved the submitted version.

## Funding

The research was supported by the National Natural Science Foundation of China (Grant Number 32002027) and the National Natural Science Foundation of Sichuan Province (Grant Number 2022NSFSC1647).

## Acknowledgments

We would like to thank Home for Researchers (www.home-for-researchers.com) for English language editing.

## Conflict of interest

The authors declare that the research was conducted in the absence of any commercial or financial relationships that could be construed as a potential conflict of interest.

## Publisher’s note

All claims expressed in this article are solely those of the authors and do not necessarily represent those of their affiliated organizations, or those of the publisher, the editors and the reviewers. Any product that may be evaluated in this article, or claim that may be made by its manufacturer, is not guaranteed or endorsed by the publisher.

## Supplementary material

Supplementary Figure S1 ModX plot of 6 celery samples and X/Y Overview Plot PCA for 10 compounds. (A) ModX plot of 6 celery samples; DModX is the distance of an observation in the training set to the x model plane or hyper plane. DModX is proportional to the residual standard deviation(RSD) of the xobservation. W: white celery; G: green celery; R: red celery; RO: root; P: petiole; L: leaf; (B)X/Y Overview Plot PCA for 10 compounds; The X/Y Overview Plot PCA shows the cumulated R2 and Q2 values for each variable of the training set(X for PCA). The 10 compounds are respectively β-Myrcene, D-Limonene, (E)-β-Ocimene, β-selinene, γ-Terpinene, Caryophyllene, Camphene, 2,4,6-Octatriene, 2,6-dimethyl-, (E,Z)-, 2-methyl-5-pentylcyclohexa-1,3-diene and 2,6-dimethyl-2,4,6-octatriene.
