# Supplementary figures and images for: Genome-wide identification and analysis of terpene synthase (TPS) genes in celery reveals their regulatory roles in terpenoid biosynthesis
Source: Front Plant Sci. 2022 Sep 29;13:1010780. doi: 10.3389/fpls.2022.1010780 (PMC9557977; doi:10.3389/fpls.2022.1010780)

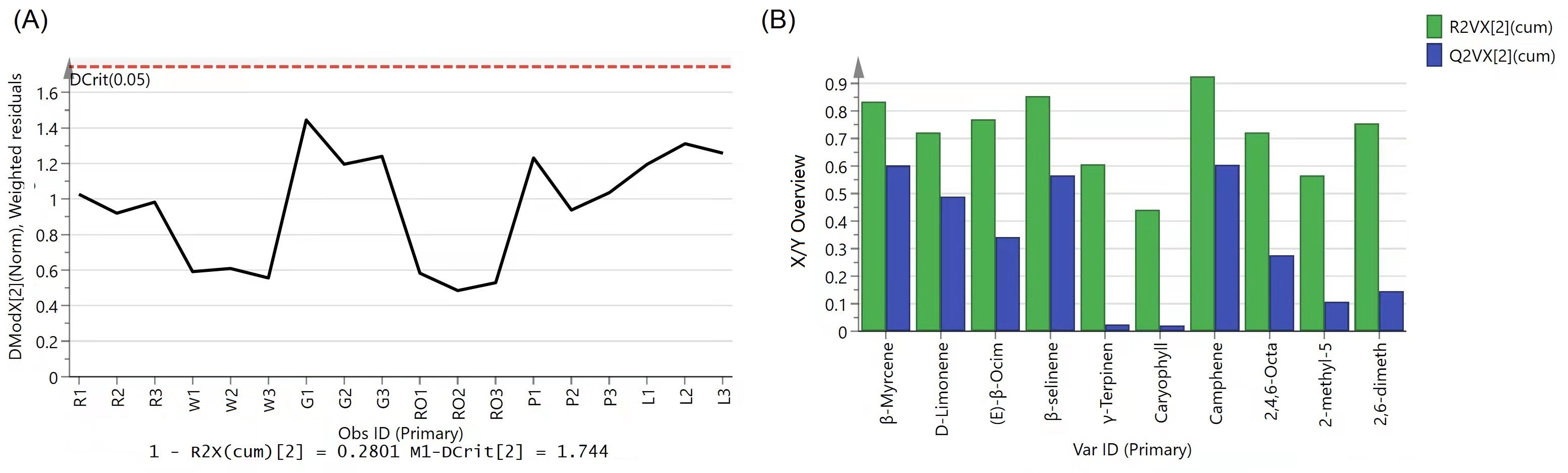

Supplement: Supplementary Figure S1 — ModX plot of 6 celery samples and X/Y Overview Plot PCA for 10 compounds. (A) ModX plot of 6 celery samples; DModX is the distance of an observation in the training set to the x model plane or hyper plane. DModX is proportional to the residual standard deviation(RSD) of the xobservation. W: white celery; G: green celery; R: red celery; RO: root; P: petiole; L: leaf; (B)X/Y Overview Plot PCA for 10 compounds; The X/Y Overview Plot PCA shows the cumulated R2 and Q2 values for each variable of the training set(X for PCA). The 10 compounds are respectively β-Myrcene, D-Limonene, (E)-β-Ocimene, β-selinene, γ-Terpinene, Caryophyllene, Camphene, 2,4,6-Octatriene, 2,6-dimethyl-, (E,Z)-, 2-methyl-5-pentylcyclohexa-1,3-diene and 2,6-dimethyl-2,4,6-octatriene. [file DataSheet_1.zip › Figure S1.jpg]
